# COVID-19 must catalyse key global natural experiments

**DOI:** 10.7189/jogh.10.010104

**Published:** 2020-06

**Authors:** Jasper V Been, Aziz Sheikh

**Affiliations:** 1 Division of Neonatology, Department of Paediatrics, Erasmus MC – Sophia Children's Hospital, University Medical Centre Rotterdam, Rotterdam, Netherlands; 2 Department of Public Health, Erasmus MC, University Medical Centre Rotterdam, Rotterdam, Netherlands; 3 Department of Obstetrics and Gynaecology, Erasmus MC – Sophia Children's Hospital, University Medical Centre Rotterdam, Rotterdam, Netherlands; 4 Centre of Medical Informatics, Usher Institute, The University of Edinburgh, Edinburgh, UK; 5 Division of General Internal Medicine and Primary Care, Brigham and Women's Hospital/Harvard Medical School, Boston, Massachusetts, USA

The COVID-19 pandemic has in a matter of weeks fundamentally crippled health systems in many countries and is now threatening to cause a global economic depression [1]. This in turn is having an unprecedented impact on health care organisations’ ability to provide emergency care and on societies to provide core functions of the state. Even in such exceptionally challenging times, it is important to search for any possible glimmers of hope. One such glimmer comes from the unique opportunity to mount global natural experiments which, if we take, could ultimately contribute to deliberations that may result in the saving of more lives than are lost in this pandemic.

We wish to highlight two key natural experiments that it is important to focus attention on. The first is countries’ responses to dealing with and mitigating the effects of the pandemic. There is considerable global variation in national responses ranging from immediate attempts to suppress the outbreak (as in China and South Korea, for example) to containment approaches aiming to promote the development of herd immunity (eg, as was initially the case in the United Kingdom and the Netherlands) [1]. These approaches will in due course need to be analysed to see which were in the round associated with the best overall population health and societal outcomes.

A second major net of natural experiments that need to be undertaken is to establish on a global scale the impact of improvements in air quality on human and planetary health. The pandemic has resulted in the greatest restrictions in travel in human history, which have already led to very substantial reductions in ambient air nitrogen dioxide and particulate matter levels [2]. Given the range of potential adverse health outcomes associated with exposure to ambient air pollution [3,4], these observed reductions may have a major positive impact on population health, particularly in highly polluted areas pre-COVID-19.

While still in the midst of the global turbulence caused by COVID-19, it is difficult to see any silver lining. We do however need to make the most of this adversity. We argue that mounting major international natural experiments to understand optimal approaches to national pandemic responses, and the effects of air quality improvements on human health represent very major opportunities to advance population health. Since such evaluations typically make use of routinely collected health care data [4-6], these efforts need not be disruptive to the immediate clinical need to address the pandemic.

We must collectively seize these opportunities to help achieve a lasting benefit of the global tragedy now unfolding.
